# Unspoken Pain: Exploring Spousal Violence and Its Associated Risk Factors Among Married Women of Reproductive Age in Rural Chengalpattu District

**DOI:** 10.7759/cureus.82234

**Published:** 2025-04-14

**Authors:** Kiruthika Narayanan, Shanthi Edward, Krishna Prasanth

**Affiliations:** 1 Department of Community Medicine, Sree Balaji Medical College and Hospital, Chennai, IND

**Keywords:** gender-based violence, intimate partner violence (ipv), rural health services, women empowerment, women reproductive health, domestic violence

## Abstract

Background

Spousal violence is a critical public health issue in India, with profound physical, psychological, and sexual consequences, particularly among married women of reproductive age in rural areas, where the risk is alarmingly high. The aim of the study is to assess various forms of spousal violence, its associated factors, and the pathways leading to different forms of abuse among women of reproductive age residing in the selected rural area of Chengalpattu District, Tamil Nadu.

Methodology

A community-based cross-sectional study was conducted on 250 married women of reproductive age. The participants were selected using systematic random sampling from a prepared line list. Data were collected from a sample of 250 women through face-to-face interviews using the Indian Family Violence and Control Scale (IFVCS), and multivariate logistic regression analysis and path analysis were performed using SPSS v25 (IBM Corp., Armonk, NY, US).

Results

Multivariate analysis identified regular alcohol consumption of husbands and spousal violence had the highest odds for all three forms of spousal violence: psychological violence (odds ratio (OR) = 5.15, 95% CI: 2.62-10.11), physical violence (OR = 6.6, 95% CI: 3.29-13.23), and sexual violence (OR = 2.89, 95% CI: 1.09-7.69). Other factors, such as nuclear families and lower socioeconomic status, were found to be significantly more vulnerable. Path analysis indicated that psychological violence often precedes physical and sexual violence, with alcohol consumption acting as a key trigger.

Conclusion

Women often face multiple interrelated forms of violence, with significant determinants including husbands’ alcohol use, nuclear families, and lower socioeconomic status. Therefore, a multifaceted strategy is needed to prevent this threat.

## Introduction

Spousal or intimate partner violence (IPV) refers to behavior by an intimate partner or ex-partner that causes physical, sexual, or psychological harm, including physical aggression, sexual coercion, psychological abuse, and controlling behavior [1]. Spousal violence against women is a significant violation of human rights and a grave public health issue. The global prevalence of sexual and spousal violence was best estimated using population-level surveys that relied on survivor reports. According to a 2018 WHO analysis of prevalence data from 2000 to 2018 across 161 countries and regions on behalf of the UN Interagency Working Group on Violence Against Women, 30% of women globally have experienced physical and/or sexual violence from an intimate partner, non-partner sexual violence, or both [1]. In 2015, members of countries agreed upon the 2030 UN Agenda for Sustainable Development Goals (SDGs), a framework that calls for the elimination of violence against women and girls under Goal 5 on gender equality and women’s empowerment [2].

Spousal violence has a profound negative impact on public health along with general and reproductive health, such as wounds, chronic pain, fractures, impairment, unintended pregnancy, and a higher incidence of sexually transmitted diseases, predominantly HIV [3]. In addition, it affects the nation’s economic growth, women’s empowerment, and their capacity to take care of themselves and others [4]. In India, according to the National Family Health Survey (NFHS) 5 survey, the overall prevalence of gender-based violence among ever-married women aged 18-49 years who have experienced spousal violence is reported to be 29.3% and 31.6% in rural India. In Tamil Nadu, 38.1% of married women experienced spousal violence and 42.2% experienced spousal violence in the rural population. In the majority of states in India, rural communities are more likely to experience spousal violence. However, when compared to NFHS 4 data, the overall prevalence of spousal violence has been reduced by a small margin, but the burden remains higher [5].

India recognized domestic violence as a criminal offense in 1983. Any act of cruelty committed by a husband (or his family) against his wife is punishable under Section 498A of the Indian Penal Code (IPC) [6]. The Protection of Women from Domestic Violence Act (PWDVA, 2005) came into force, considering the multifaceted nature of certain domestic abuse cases. According to PWDVA (2005), domestic violence includes both real acts of violence and threats and encompasses all types of physical, sexual, verbal, emotional, and economic violence [7]. Spousal violence, domestic violence, or IPV is a preventable cause of mortality and morbidity in women [8]. With this context in mind, understanding the multifactorial nature of spousal violence is essential not only for academic exploration but also for shaping effective public health responses. The findings of this study are anticipated to serve as crucial evidence for policymakers and community-based organizations to inform their efforts in addressing violence in rural areas. Hence, this study aims to assess the various forms of spousal violence, its associated factors, and the pathways leading to different forms of abuse among the reproductive age group of women residing in selected rural areas in the Chengalpattu district of Tamil Nadu.

## Materials and methods

Study setting

A community-based cross-sectional study was conducted among women of reproductive age residing in rural areas in Chengalpattu District, Tamil Nadu. Chengalpattu District was selected for this study due to its demographic heterogeneity, accessibility, and operational feasibility, as the research team is institutionally affiliated within the same region, and due to underrepresentation in the literature. The total duration of the study was six months, from July 2024 to December 2024.

Inclusion and exclusion criteria

All married women of reproductive age, who gave complete responses to the entire questionnaire, and participants who understood and communicated in Tamil or English, as the questionnaire was administered in these languages, were included in the study. Newly married women less than one year of marriage, women who are temporary visitors to visit parents/relatives, and those who are reluctant to participate in the study were excluded.

Sample size calculation

The sample size was calculated based on the NFHS 5 survey with the prevalence of spousal violence among women of reproductive age residing in rural India (31.6%) [5]. Using this as prevalence (p) and substituting it in Dapson’s formula and adding a 5% non-response rate, the final sample size of 250 was obtained. One participant did not fill out the questionnaire completely and, hence, was excluded. A total of 249 participants were included in the study.

Sampling technique

**Figure 1:**
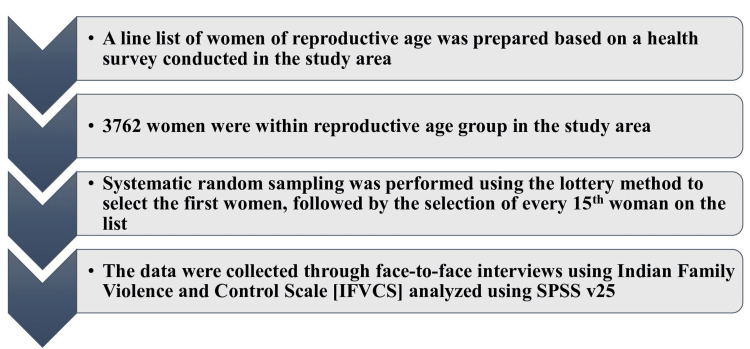
Sampling methodology

To identify the sample population, a line list of women of reproductive age was prepared based on a health survey conducted in the study area. In the study area, 3,762 women were within the reproductive age group, and the sampling interval was calculated to be 15. Systematic random sampling was performed using the lottery method to select the first women, followed by the selection of every 15th woman on the list. If a woman was not available, two additional visits were scheduled on different days, and if not available, the next woman on the list was included (Figure 1).

Data collection tool

The data were collected through face-to-face interviews, conducted by trained female postgraduates from our department, focusing on ethical considerations and methods to ensure respondent confidentiality and comfort. Interviews were conducted in private settings to maintain confidentiality, and participants were assured of anonymity and voluntary participation. The Indian Family Violence and Control Scale (IFVCS) [9], which is a 63-item questionnaire including physical, psychological, and sexual factors, along with sociodemographic information was included.

Data analysis

Statistical analysis of this study includes the chi-squared test to assess associations, multivariate logistic regression was done to determine the odds ratio (OR) for risk factors, and path analysis found the escalation patterns between different forms of spousal violence. Analysis was performed using SPSS v25 (IBM Corp., Armonk, NY, US). No missing data were encountered in the final dataset, and model assumptions and interactions were checked prior to logistic regression and path analysis.

## Results

Data were obtained from 249 women in the reproductive age group. The majority of study participants were in the age group 18-30 years (147 (59%)). The study found that there was a higher proportion of literate husbands than their wives (46.2% vs. 41%), and unemployment was higher among women (130 (52.2%)). Socioeconomic status was assessed using the B. G. Prasad scale [10], with the lower middle class being the most represented (109 (43.8%)), followed by the middle class (73 (29.3%)), lower class (42 (16.9%)), upper middle class (20 (8%)), and upper class (5 (2%)). More than half of the study population belongs to the nuclear family, 134 (53.8%), in comparison with the joint family, 115 (46.2%). A large proportion of husbands consumed alcohol on a regular basis (156 (62.7%)) (Table 1).

**Table 1:** Demographic characteristics of the participants (n = 249)

Variables	Frequency (%)
Age of women (years)	
18-30	147 (59%)
31-49	102 (41%)
Literacy of husband	
Illiterate	134 (53.8%)
Literate	115 (46.2%)
Literacy of women	
Illiterate	147 (59%)
Literate	102 (41%)
Occupation of women	
Unemployed	130 (52.2%)
Employed	119 (47.8%)
Socioeconomic status	
Upper class	5 (2%)
Upper middle class	20 (8%)
Middle class	73 (29.3%)
Lower middle class	109 (43.8%)
Lower class	42 (16.9%)
Family status	
Nuclear	134 (53.8%)
Joint	115 (46.2%)
Regular alcohol consumption of husband	
Yes	156 (62.7%)
No	93 (37.3%)

Table 2 describes the associations between different forms of spousal violence and its related variables. The chi-squared analysis was performed to assess the association. The association between age and spousal violence indicated that age was not significantly associated with any form of violence. The p-values for psychological (p = 0.7165), physical (p = 0.2187), and sexual (p = 0.6754) violence suggest no significant differences in the prevalence of violence among the different age groups. However, a slightly higher proportion of younger women (18-30 years) reported experiencing both psychological and physical violence than older women (31-49 years). Among women aged 18-30 years, 22 (15.0%) reported experiencing psychological violence approximately once a month, while 10.2% experienced physical violence at the same frequency. In contrast, sexual violence was reported at lower rates in both age groups, with more than 84% of women in each category reporting that they had never experienced it.

**Table 2:** Chi-squared analysis-association between different forms of violence and its related variables *Statistically significant p-values

Variable		Spousal violence					
	Category	About once a month	About once a week	About once or twice in the past year	Never	Not in the past year but it did happen before in my married life	p-value
Age vs. psychological violence	18-30	22 (15.0%)	11 (7.5%)	24 (16.3%)	88 (59.9%)	2 (1.4%)	0.716
	31-49	14 (13.7%)	9 (8.8%)	14 (13.7%)	61 (59.8%)	4 (3.9%)	
Age vs. physical violence	18-30	15 (10.2%)	12 (8.2%)	27 (18.4%)	93 (63.3%)	0 (0.0%)	0.218
	31-49	14 (13.7%)	9 (8.8%)	20 (19.6%)	56 (54.9%)	3 (2.9%)	
Age vs. sexual violence	18-30	3 (2.0%)	1 (0.7%)	15 (10.2%)	128 (87.1%)	0 (0.0%)	0.675
	31-49	1 (1.0%)	1 (1.0%)	13 (12.7%)	86 (84.3%)	1 (1.0%)	
Socioeconomic status vs. psychological violence	Lower	8 (19.0%)	4 (9.5%)	2 (4.8%)	28 (66.7%)	0 (0.0%)	0.047*
	Lower middle class	21 (19.3%)	11 (10.1%)	17 (15.6%)	58 (53.2%)	2 (1.8%)	

The present study found a statistically significant association between socioeconomic status and psychological violence (p = 0.0475), with lower and middle class women experiencing higher levels of violence. In the lower socioeconomic group, eight (19.0%) women reported psychological violence occurring about once a month, while in the lower middle class group, the prevalence was 21 (19.3%). However, the highest frequency of physical violence was observed in the middle class group, where 10 (13.7%) women experienced violence about once a month. Sexual violence was not significantly associated with socioeconomic status, as indicated by chi-squared analysis.

In Table 3, regular alcohol consumption by the spouse was found to have a highly significant association with both psychological violence (p = 0.0001) and physical violence (p = 0.0008). Women whose husbands regularly consumed alcohol reported significantly higher rates of both psychological and physical violence compared to those whose spouses did not drink. Among women whose spouses consumed alcohol, 31 (19.9%) experienced psychological violence about once a month, and 28 (17.9%) faced physical violence with the same frequency. In contrast, among women whose spouses did not consume alcohol, only five (5.4%) reported psychological violence and one (1.1%) reported physical violence occurring once a month. This finding suggests that alcohol consumption is a major risk factor for IPV.

**Table 3:** Chi-squared analysis-association between different forms of violence and its related variables *Statistically significant p-values

Variable		Spousal violence					
	Category	About once a month	About once a week	About once or twice in the past year	Never	Not in the past year but it did happen before in my married life	p-value
Regular alcohol consumption vs. psychological violence	No	5 (5.4%)	2 (2.2%)	8 (8.6%)	73 (78.5%)	5 (5.4%)	0.0001*
	Yes	31 (19.9%)	18 (11.5%)	30 (19.2%)	76 (48.7%)	1 (0.6%)	
Regular alcohol consumption vs. physical violence	No	1 (1.1%)	3 (3.2%)	11 (11.8%)	75 (80.6%)	3 (3.2%)	0.0008*
	Yes	28 (17.9%)	18 (11.5%)	36 (23.1%)	74 (47.4%)	0 (0.0%)	
Family status vs. psychological violence	Joint	8 (7.0%)	7 (6.1%)	16 (13.9%)	82 (71.3%)	2 (1.7%)	0.0054*
	Nuclear	28 (20.9%)	13 (9.7%)	22 (16.4%)	67 (50.0%)	4 (3.0%)	
Family status vs. physical violence	Joint	12 (10.4%)	12 (10.4%)	14 (12.2%)	76 (66.1%)	1 (0.9%)	0.0929
	Nuclear	17 (12.7%)	9 (6.7%)	33 (24.6%)	73 (54.5%)	2 (1.5%)	

This study identified a statistically significant association between family structure and psychological violence (p = 0.0054). Women living in nuclear families reported significantly higher rates of psychological violence than those living in joint families. Specifically, 28 (20.9%) women in nuclear families experienced psychological violence about once a month, whereas only eight (7.0%) women in joint families reported the same. However, physical violence did not show a statistically significant association with family structure (p = 0.0929). However, nuclear families reported slightly higher rates of physical violence. Family status had a greater impact on spousal violence.

In Table 4, logistic regression analysis revealed that women whose husbands consume alcohol regularly were at significantly higher risk of experiencing all three forms of spousal violence-psychological violence (OR = 5.15, 95% CI: 2.62-10.11), physical violence (OR = 6.6, 95% CI: 3.29-13.23), and sexual violence (OR = 2.89, 95% CI: 1.09-7.69). A nuclear type of family was associated with an increased risk of experiencing psychological violence (OR = 3.38, 95% CI: 1.87-6.1). Women belonging to lower socioeconomic status were significantly associated with physical violence (OR = 1.89, 95% CI: 1.03-3.48).

**Table 4:** Logistic regression analysis between different forms of violence and its associated variables *Statistically significant p-values

Variables	Odds ratio	95% CI	p-value
A. Psychological violence			
Regular alcohol consumption of husband (yes vs. no)	5.15	2.62-10.11	<0.0001*
Family type (nuclear vs. joint)	3.38	1.87-6.1	0.0001*
B. Physical violence			
Regular alcohol consumption of husband (yes vs. no)	6.6	3.29-13.23	<0.0001*
Socioeconomic status (higher vs. lower SES)	1.89	1.03-3.48	0.0394
Family type (nuclear vs. joint)	2.08	1.17-3.72	0.0129
C. Sexual violence			
Regular alcohol consumption of husband (yes vs. no)	2.89	1.09-7.69	0.0333*

**Figure 2:**
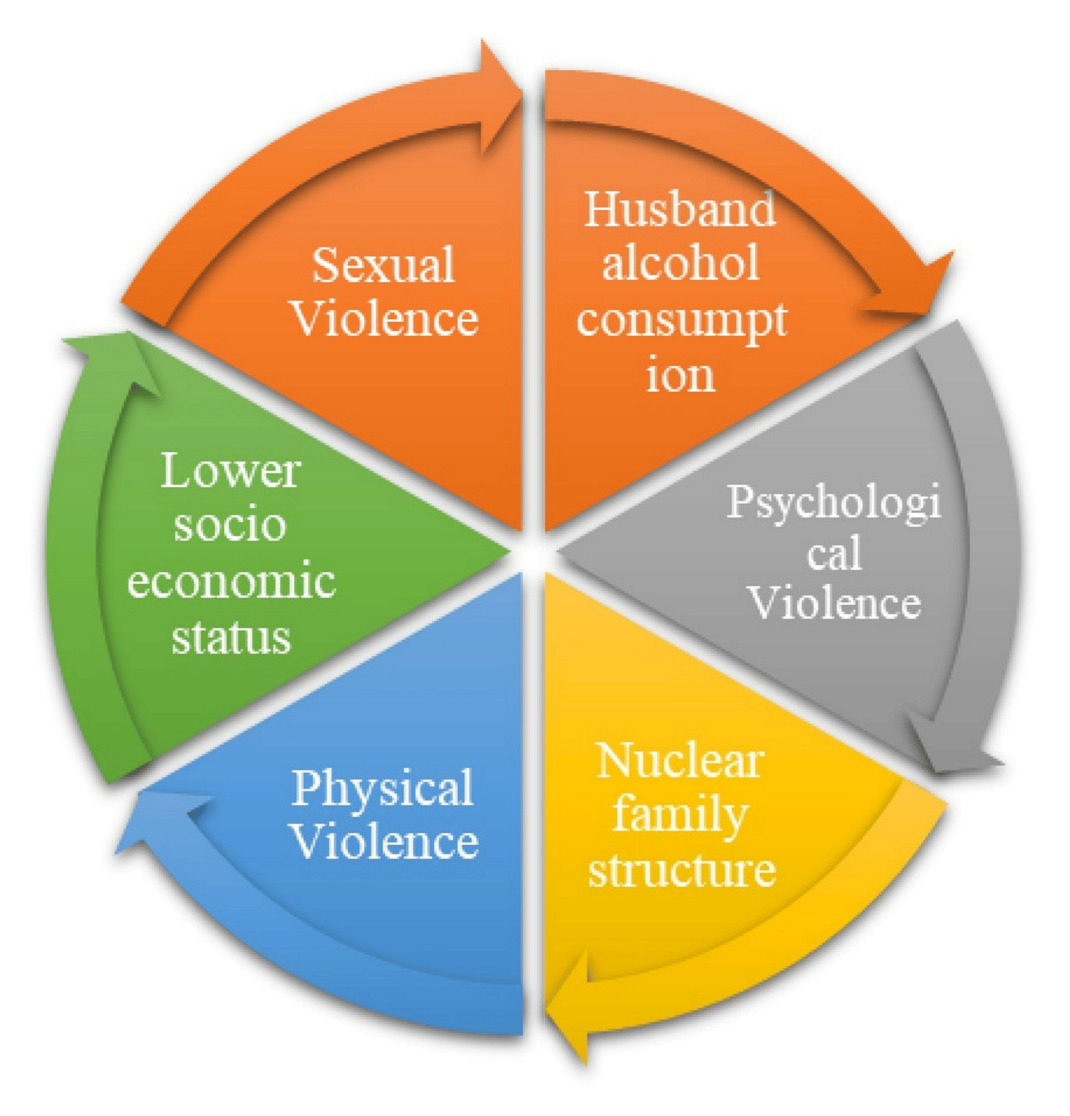
Path analysis of various forms of spousal violence The image is Dr. Kiruthika Narayanan's original creation, developed specifically for this research work. It has not been reproduced, reprinted, or adapted from any previously published source.

Figure 2 describes the path analysis of different forms of spousal violence and its associated factors leading to another form of violence. Husbands’ regular alcohol consumption is identified as a common risk factor influencing all three forms of violence: psychological, physical, and sexual violence. Psychological violence is directly influenced by the husband's alcohol consumption and nuclear family structure. Women in nuclear families may have limited social support, making them more vulnerable to emotional abuse, verbal threats, and controlling behaviors by their spouses. Psychological violence often serves as a precursor to physical violence, leading to a gradual escalation of abuse over time. Physical violence is influenced by the husband's alcohol consumption, lower socioeconomic status, and nuclear family structure. Financial stress in lower socioeconomic groups may increase family tensions, leading to domestic conflicts that escalate into physical violence. Women in nuclear families may face a higher risk due to isolation from extended family support. Eventually, women who are experiencing physical violence due to alcohol consumption by their husbands, belonging to lower socioeconomic status, and living in a nuclear type of family have more risk of leading to sexual violence. Sexual violence is primarily influenced by the husband's alcohol consumption. Alcohol impairs judgment and increases aggressive sexual behavior, leading to non-consensual or forced sexual acts within marriage. The arrows in Figure 2 are intentionally unidirectional to represent hypothesized causal pathways derived from literature and supported by the path analysis model. While some relationships may be bidirectional in real-world scenarios, the model was structured to reflect the most statistically supported direction of influence.

## Discussion

The present study highlights multiple interrelated forms of spousal violence: psychological, physical, and sexual, among married women of reproductive age in rural Chengalpattu District. The findings reveal that women residing in nuclear families, belonging to lower socioeconomic strata, and whose spouses regularly consume alcohol are at increased vulnerability. These associations underscore the multifactorial and intersecting nature of IPV in rural Indian settings. The results also bring attention to underexplored social determinants and help inform future strategies to mitigate such violence at the household and community levels.

Spousal violence is significantly influenced by women’s age, particularly in the context of marriage. In this present study, no significant association was found between age and spousal violence. However, a slightly higher proportion of younger women (18-30 years) reported experiencing both psychological and physical violence than older women (31-49 years). Similarly, a study conducted in Nagpur, India, found that younger women reported a higher prevalence of spousal violence [11]. Another study found that younger women, especially those who were married before turning 18 years, were more vulnerable. Research shows that IPV rates are much greater for those who get married at age 15 than for those who get married at age 24 [12]. In contrast, a study done by Krishnamoorthy et al. found that older women are more likely to be subjected to physical, mental, and sexual abuse by their partners [13]. This difference could be due to a combination of actual differences in violence patterns, variations in reporting behavior, and sociocultural differences across regions.

A delicate and complex link exists between literacy and spousal violence. The present study found that the literacy rate (41%) was lower among married women than among their husbands (46%). Similar results were found in other studies; spousal violence is strongly impacted by the literacy rate, with lower-educated or illiterate couples reporting higher rates of spousal violence. The same trend is observed in lower- and middle-income countries, where lower levels of education are associated with higher tolerance of IPV [14,15].

Lower socioeconomic status often correlates with increased vulnerability to IPV, as economic stressors can exacerbate tensions within relationships. The present study found that married women who belonged to lower socioeconomic status had higher odds of experiencing the physical form of spousal violence (OR 1.89, 95% CI: 1.03-3.48). Similarly, various other studies conducted in India found that physical violence and psychological violence are more common among women from lower socioeconomic classes, and there is a significant association between families with lower income and an increasing rate of violence [16-18]. Likewise, a study conducted in China found that men who belong to lower socioeconomic status are more prone to commit domestic violence due to paternity uncertainty when compared to men of higher socioeconomic status [19]. Increased IPV is linked to resource inequality, namely, in terms of income and decision-making authority, notably in areas such as South Asia and East-Southern Africa [20]. In contrast, a study conducted in India found that women with higher socioeconomic status may paradoxically be more likely to experience domestic violence because they may be subjected to more controlling behavior from their spouses, which reflects power relations and financial hardship and can escalate violence [21]. Spousal violence in India among women of lower socioeconomic status often stems from financial stress, lack of education, and social norms that tolerate abuse. Conversely, in higher socioeconomic groups, violence may arise due to power dynamics, emotional abuse, or controlling behavior masked by social prestige, limiting the victims' ability to seek help.

The present study found that women belonging to the nuclear type of family had the highest odds of experiencing psychological and physical spousal violence (OR 3.38, 95% CI: 1.87-6.1). Likewise, a study conducted in rural areas of Maharashtra found that more than two-thirds of women facing domestic violence belong to nuclear families [22], and a study conducted in rural Gujarat had similar findings [23]. The risk of spousal violence is higher among nuclear family members, probably because of a lack of support from elderly family members, which makes them more vulnerable. In contrast, a study conducted in New Delhi states that spousal violence is significantly associated with a joint family [24].

In the present study, husbands who consumed alcohol on a regular basis had the highest odds of women experiencing spousal violence in all forms, among which the highest being physical violence (OR 6.6, 95% CI: 3.29-13.23). A comparable study conducted in Tamil Nadu found that the highest prevalence of spousal violence was related to alcohol consumption [25]. Similarly, studies conducted in Eastern India and Goa have found that regular alcohol consumption by husbands significantly increases the risk of women experiencing different forms of spousal violence [26,27]. The relationship between spousal violence and the employment status of married women is intertwined. In the present study conducted in a rural setting, more than half of the married women were unemployed. Particularly in India, working women are more likely to be victims of spousal violence, probably due to the prevalence of traditional gender roles and financial exploitation [28].

In this study, no association was found between spousal violence and dowry. Interestingly, contradicting the findings of the present study, certain studies conducted in India have found an association between spousal violence and dowry. A study done by Mani et al. found that dowry demand had an indirect impact on psychological violence by controlling for variables including alcohol use and social support, suggesting a strong correlation between dowry and spousal violence [29]. Similarly, a study conducted in Uttar Pradesh and Bihar found that younger women who were demanded dowry by their in-laws had higher odds of experiencing any form of violence (adjusted OR (AOR): 3.66; CI: 3.06-4.37) [30].

Limitation

It is important to consider the limitations of this study to properly understand the results. Spousal violence is a sensitive issue, especially in rural areas, where social norms may discourage women from openly discussing such experiences, which can lead to substantial underreporting. As the study relied on self-reports, the data may be subjective and influenced by social desirability bias. Women may provide responses that they believe are socially acceptable rather than truthful accounts, and despite efforts to ensure anonymity, participants may still feel unsafe or fear potential exposure, which may lead to information bias. Participants may struggle to accurately recall details about incidents of violence, especially if the events occurred long ago. The absence of triangulation methods such as qualitative interviews limits the interpretive depth of the findings.

Recommendation

Thus, a multifaceted strategy is required to prevent imminent threats. Awareness campaigns should be conducted to educate both men and women about the legal rights of women, the consequences of domestic violence, and available support services. Strengthening the roles of local self-help groups, Anganwadi workers, and Accredited Social Health Activist (ASHA) workers can help identify and support victims effectively. Community-based counseling services, crisis helplines, and safe shelter facilities should be expanded to offer immediate support. Women in rural areas should be educated on the harmful effects of alcohol consumption to prevent spousal violence. Empowering women through skill development programs, education, and financial independence initiatives can reduce their vulnerability. A multisectoral collaboration involving healthcare providers, law enforcement, and non-governmental organizations is essential for creating a comprehensive support system. Further research should focus on understanding the sociocultural determinants of violence to develop context-specific interventions for lasting change.

## Conclusions

This study helped to explore the burden of various forms of spousal violence in rural areas among married women of reproductive age. Each form of spousal violence is deeply interrelated, and most women experience multiple forms of spousal violence in their marriages. Although the physical form of violence is more apparent, many women experience psychological and sexual forms of violence, which may impact their health. Regular alcohol consumption by husbands, residing in nuclear families, and belonging to a lower socioeconomic class were important determinants of spousal violence and were found to be statistically significant. The path analysis shows how different forms of violence may be linked, but these findings should be viewed with caution.
